# Influenza A Infection and Stem Cell Mobilization

**DOI:** 10.4274/tjh.2018.0089

**Published:** 2018-05-25

**Authors:** Sora Yasri, Viroj Wiwanitkit

**Affiliations:** 1 KMT Primary Care Center, Bangkok, Thailand; 2 Hainan Medical University, Department of Tropical Medicine, Haikou, Hainan, China

**Keywords:** Influenza, Infection, Stem cell

## To the Editor,

We read the publication entitled “Use of Plerixafor to Mobilize a Healthy Donor Infected with Influenza A” and found it to be very interesting [1]. Yeral et al. [1] mentioned that “The effects of influenza A infection on stem cell mobilization are not known” and concluded that “This report indicates that influenza A may suppress the hematopoietic system, negatively affecting stem cell mobilization. The problem may be overcome by plerixafor administration” [1]. This article may provide a new observation and confirm the usefulness of plerixafor in achieving stem cell mobilization. Nevertheless, it should be noted that this is not the first case of stem cell transplantation in which the donor has influenza A infection. Lee et al. [2] reported stem cell transplantation from a related donor infected with influenza H1N1 2009 and in that case the transplantation was completely done without noting any problem of stem cell mobilization due to the influenza virus. Regardless of using plerixafor, however, stem cell transplantation in cases in which the donor has influenza infection is a considerable challenge and it is questionable whether the procedure should be done then or not.
