# Supplementary material for: Influenza A Infection and Stem Cell Mobilization
Source: Turk J Haematol. 2018 May 25;35(2):139–40. doi: 10.4274/tjh.2018.0089 (PMC5972340; doi:10.4274/tjh.2018.0089)
Supplement: Related texts

# Reply to the Authors:

**Authors:** Mahmut Yeral, Pelin Aytan, Can Boğa

## To the Editor,

We read the recent letter by Yasri and Wiwanitkit [1] regarding our manuscript with great interest. We are pleased with their contributions and comments. The literature data with regard to the effect of influenza A on hematopoietic cell mobilization is limited to only several case reports [2,3].

However it would not be incorrect to relate mobilization failure to Influenza in a donor who has no prior diseases, who is not using any kind of medication or substance, and who is considered to be healthy in clinical and laboratory evaluations before mobilization. Mobilization failure may be associated with cytokine increase, presence of viremia and viral titers. Lee et al. [2] reported three donors who were infected with influenza. Mobilization was postponed for a short period of time in one donor due to poor mobilization risk. Two donors could be mobilized with granulocyte-colony stimulating factor. Nevertheless one of the donors could not be regarded as good mobilized. Because despite two days of apheresis procedure, the collected CD34+ cells from the healthy donor were ≤2×10 6/kg.

With our case we aimed to point out that influenza A may affect mobilization negatively and this condition may be overcome with plerixafor. It should be known that mortality is inevitable in a patient who received myeloablative conditioning regimen without stem cells.

## References

[1] Yasri S, Wiwanitkit V (2018). Influenza A Infection and Stem Cell Mobilization. Turk J Hematol.

[2] Lee SH, Cheuh H, Yoo KH, Kim YJ, Sung KW, Koo HH, Kim DH, Kim SJ, Kim K, Jang JH, Jung CW (2011). Hematopoietic stem cell transplantation from a related donor infected with influenza H1N1 2009. Transpl Infect Dis.

[3] Rohn A, Kessler HH, Valentin T, Linkesch W, Neumeister P (2011). Prophylactic oseltamivir treatment for prevention of donor-recipient influenza A H1N1 virus transmission does not compromise stem cell mobilization or engraftment. Bone Marrow Transplant.
